# Loratadine-associated cystoid macular edema: A case report

**DOI:** 10.1016/j.ajoc.2022.101477

**Published:** 2022-03-09

**Authors:** Yong Tang, Rui Dou, Yuyan Liu, Shiyong Xie, Quanhong Han

**Affiliations:** Tianjin Eye Hospital, Tianjin Key Lab of Ophthalmology and Visual Science, Tianjin Eye Institute, Nankai University Affiliated Eye Hospital, Clinical College of Ophthalmology, Tianjin Medical University, Tianjin, China

**Keywords:** Cystoid macular edema, Loratadine, Optical coherence tomography, Histamine

## Abstract

**Purpose:**

To report the first known case of bilateral cystoid macular edema in a patient undergoing long-term loratadine treatment.

**Observations:**

A 49-year-old Chinese woman who had been undergoing treatment with loratadine for the past 6 years presented with decreased visual acuity and bilateral cystoid macular edema (CME). Upon cessation of loratadine, macular edema partially resolved, and visual acuity markedly improved. Fundus autofluorescence (FAF), optical coherence tomography (OCT), and fluorescence fundus angiography (FFA) were used to document the severity of CME and its subsequent resolution after cessation of loratadine therapy.

**Conclusions and Importance:**

Long-term use of loratadine might cause CME that partially resolves with discontinuation of the drug. The pathophysiology of drug-induced CME without leakage remains unclear. Dysfunction of histamine receptor1-expressed retinal neurons and the associated signal transduction, toxicity to Müller cells or RPE cells with subsequent intracellular fluid accumulation, and subclinical damage to the blood-retina barrier leading to leakage of extracellular fluid, have been proposed.

## 1 Introduction

Loratadine, a selective inverse agonist of peripheral histamine H1 receptors, is used to relieve the symptoms associated with allergic rhinitis and seasonal allergic conjunctivitis.^1^ It is devoid of significant central and autonomic nervous system effects, and extensive clinical investigations have proven the safety and efficacy of loratadine in the treatment of allergic symptoms.^1^ To date, at the recommended doses, the ocular side effects of loratadine have been reported only on ocular drying.^2^^,^^3^ In the present report, we describe the first case of bilateral cystoid macular edema (CME) in a Chinese woman with allergic rhinitis who underwent loratadine therapy for six years, before presenting to our hospital.

## 2 Case report

A 49-year-old Chinese woman complained of gradually blurring vision in her right eye for the past one year. She had been diagnosed with allergic rhinitis eight years ago and received loratadine 5 mg per day routinely for the past six years. The patient had no history of diabetes, hypertension, or ocular diseases, and had not yet undergone any intraocular surgery. Her family history was negative for congenital X-linked retinoschisis, Goldmann-Favre syndrome, and retinitis pigmentosa. She was not taking any other drugs and had no conditions associated with macular damage.

**Fig. 1:**
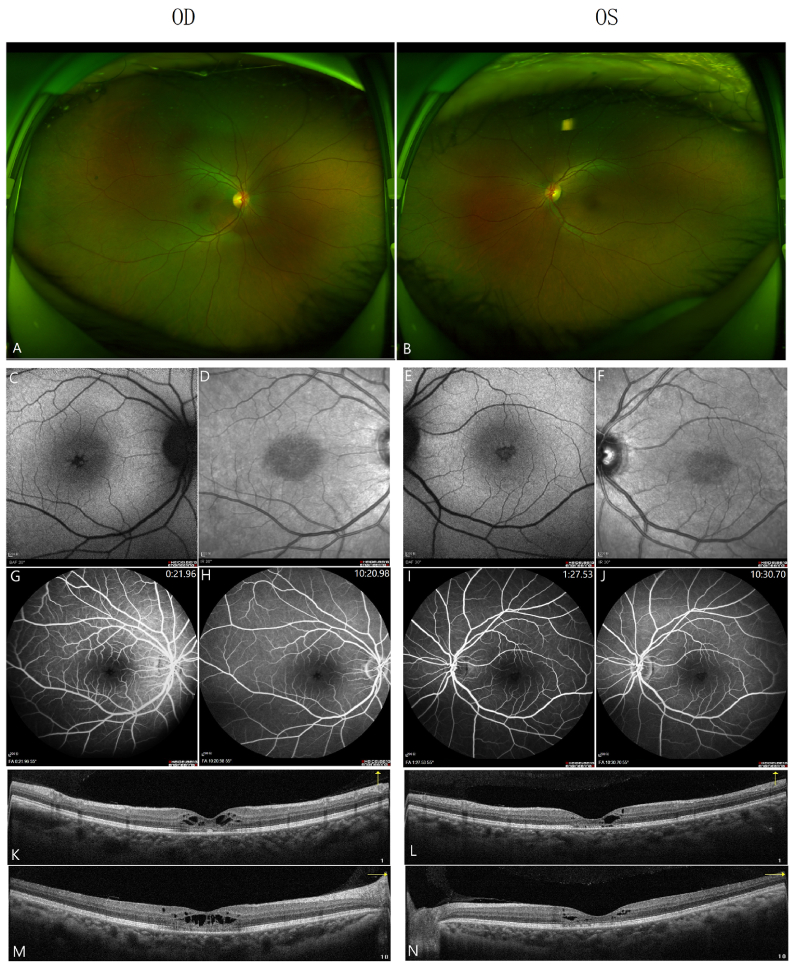
Multimodal images of both eyes (OD/OS) at initial examination. (A, B) Retinal blood vessels were normal with no edema, bleeding or exudation as observed in wide-field fundus photograph. No definite fovea reflections bilaterally. (C, E) BAF reveals stellate appearance of the fovea bilaterally with no evidence of posterior segment inflammation or optic disc hyperfluorescence. (D, F) IR AF shows abnormal areas of hyporeflectance in the central macula. (G–J) FFA shows stellate appearance of the fovea bilaterally with no evidence of vascular leakage or optic disc hyperfluorescence. (K–N) OCT shows intraretinal cystic changes, mainly affecting the outer plexiform layer (OPL) and outer nuclear layer (ONL) with ellipsoid zone disruption. BAF, Blue-light Fundus autofluorescece; IR AF, Near-infrared autofluorescence; FFA, fundus fluorescence angiography; OCT, ocular contrast tomography. (For interpretation of the references to colour in this figure legend, the reader is referred to the Web version of this article.)

At the initial ophthalmologic examination, the best-corrected visual acuity was 3/20 OD and 8/20 OS, and intraocular pressure was 18 mmHg OD and 17 mmHg OS. The anterior segment examination was unremarkable, except for incipient nuclear sclerotic cataract. Pupils, extraocular motility, and confrontation visual fields were within normal limits. Funduscopic examination revealed healthy optic nerves and vessels along with a normal peripheral fundus, aside from absence of definite foveal light reflex in either eye. (Fig. 1A and B; Optomap 200Tx, Optos, UK). Posterior vitreous detachment was noted bilaterally with no evidence of vitreoretinal interface abnormalities. Fundus autofluorescence (FAF) (Fig. 1C–F) revealed bilateral stellate hypofluorescence in the fovea. Fluorescein angiograms (Fig. 1G–J, Heidelberg Spectralis HRA + OCT, Heidelberg Engineering GmbH, Heidelberg, Germany) revealed normal filling of the choroidal and retinal vessels and an intact parafoveal capillary net. The late frames of the angiograms did not show any significant vascular leakage.RTVue optical coherence tomography (OCT) (Optovue Inc., Fremont, CA) revealed bilateral CME with cystoid changes in the outer plexiform and inner nuclear layers (Fig. 1K-N).

In the present case, chronic progression of signs and symptoms with loratadine and bilateral CME was observed. To the best of our knowledge, this is the first case of bilateral CME associated with loratadine therapy. Considering that the patient did not take any other drugs or present with other medical conditions associated with macular edema, CME was thought to be secondary to loratadine use. After consultation with an otolaryngologist, loratadine therapy was discontinued.

**Fig. 2:**
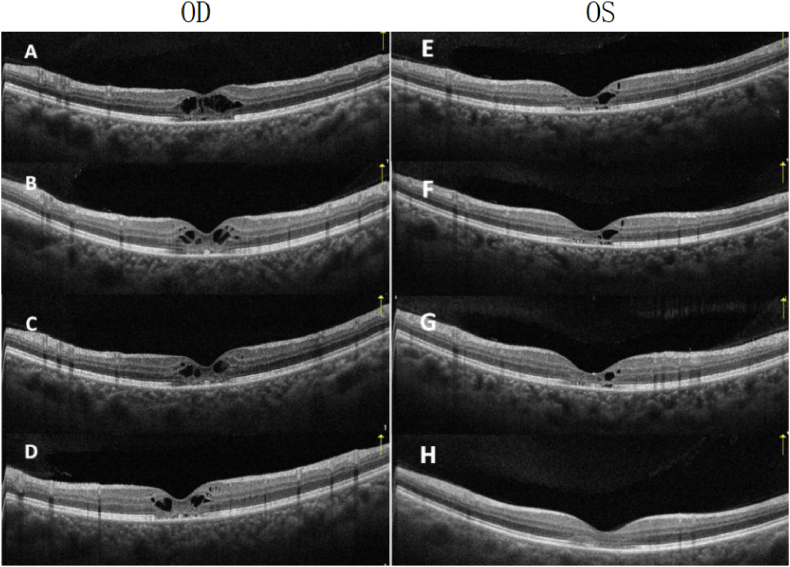
OCT changes in both eyes. (A,E) Baseline examination. Intraretinal cystic changes in fovea, mainly affect IPL and OPL with discontinuity of outer retinal layers. (B) Changes at one month after cessation of loratadine; macular cystic changes partially resolved. (C) Two months after cessation of loratadine; continued resolution of intraretinal cysts and outer retinal layers. (D) Six months after cessation of loratadine; residual intraretinal cysts still observed in OD. Complete restoration of intraretinal layers and partial recovery of outer layers in OS. IPL, inner plexiform layer; OPL, outer plexiform layer; OCT, ocular computed tomography.

Four weeks after the initial visit, the patient was symptomatically better and her BCVA improved to 12/20 OD and 18/20 OS, with bilateral structural improvement of macular edema (Fig. 2 B,F).

At 2-month follow-up, her BCVA had further improved to 16/20 OD and 20/20 OS. CME partially resolved in OD (Fig. 2C) and markedly improved in OS (Fig. 2G).

At 6-month follow-up, BCVA was 18/20 OD and 20/20 OS. There were some residual macular cystoid spaces in OD (Fig. 2D). CME was fully resolved with complete restoration of visual acuity and foveal structure in OS (Fig. 2H). with some residual macular cystoid spaces in OD (Fig. 2D).

## 3 Discussion

Drug-induced CME can be caused by various drugs such as taxanes,^4^ acitretin^5^, tamoxifen,^6^ niacin^7^, interferon, fingolimod,^8^ prostaglandin, epinephrine, and timolol among others.^9^ However, systemic anti-histamines- or anti-histamine eye drops-induced CME has not yet been reported. To date, this is the first case of bilateral CME associated with loratadine therapy.

CME is characterized by abnormal thickening of the retina associated with excess fluid accumulation within the macular retina following disruption of the blood-retinal barrier. Furthermore, Müller cells play an important role in macular dehydration via metabolic pumps.^10^ These conditions are induced by microvascular occlusion or underlying inflammatory disease.^11^

The typical fluorescein angiographic appearance of CME consists of small focal leaks that increase in size and intensity early on, resulting in late pooling and a characteristic flower-petal pattern with or without leakage surrounding the optic nerve.^1^ While for drug-induced CME, several papers have reported that CME was not observed to be associated with leakage on fluorescein angiography.^12^, ^13^, ^14^, ^15^ Therefore, the mechanisms of drug-induced CME may differ from those of microvascular occlusion or inflammatory disease. The characteristics of drug-induced CME^6^ are: (1) often bilateral, (2) no leakage on fluorescence fundus angiography (FFA), (3) a larger cystic space in the outer rather than the inner layer of the retina on OCT scans, and (4) spontaneous resolution and improvement of visual acuity after discontinuation of the causative drug.

Several questions need to be answered before we can deduce that loratadine might be a potential cause of drug-induced CME in this patient:

1) Whether loratadine can pass the blood-retinal barrier (BRB)?
2) Whether there are Histamine (HA) neurons in the retina?
3) Whether there are histamine receptors in the retina?

As a second-generation antihistamine, loratadine is a reverse agonist of peripheral histamine receptor 1(HR1). The sedating effect of first-generation H1-antihistamines has been associated with the penetration of the blood-brain barrier (BBB) and lack of efflux by *P*-glycoprotein (Pgp).^16^ Some second-generation antihistamines, such as terfenadine and loratadine, can cause dose-dependent sedation, indicating their potential to cross the BBB.^17^ Lack of sedative effects on the central nervous system (CNS) and restricted BBB penetration have been proposed to arise from Pgp-mediated efflux.^18^ Given the structural similarities between the BBB and BRB, there might be potential pathways for loratadine to gain access to the retina. However, little is known about the penetration of loratadine across the BRB.

Histamine (HA), a small molecule synthesized from the amino acid histidine, is associated with allergy, inflammation, and T-cell regulation.^19^^,^^20^ In the brain, histamine is stored in mast cells and other non-neuronal cells and acts as a neurotransmitter.^21^^,^^22^ The firing patterns of HA neurons show circadian rhythms,^23^ and HA is known to promote arousal, which is consistent with the drowsiness effects of antihistamines. HA neuron cell bodies lie in the tuberomammillary (TM) nucleus of the hypothalamus, and these neurons send projections throughout the CNS, particularly to the cerebral cortex, retina, amygdala, basal ganglia, hippocampus, thalamus, and spinal cord.^24^ Mast cells, which are another possible source of histamine, are not present in the retina.

Projected from the TM nucleus in the hypothalamus, the functions of retinopetal axons in mammalian retina is not well understood. HA has been localized to retinopetal axons in the guinea pig,^25^ monkey,^26^ and rat^27^ retinas. The axons of these neurons enter the retina through the optic disc, run through the optic fiber layer, and terminate in the inner plexiform layer (IPL).^28^

Using immunofluorescence, previous research^29^ has shown that HR1 is expressed by horizontal cells and a small number of amacrine cells. HR2 is closely associated with the synaptic ribbons inside the cone pedicles. HR3 was located on the tips of the ON bipolar cell dendrites. All three major targets of histamine^23^ are distributed in the outer plexiform layer, while retinopetal axons containing histamine terminate in the inner plexiform layer. These findings suggest that the effects of histamine released by the retinopetal axons in the primate retina are mediated by volume transmission. Inhibition by an HR antagonist might interfere with signal transduction or cellular metabolism of related retinal neurons and lead to subsequent intracellular edema. Other possibilities have been proposed to explain the etiology of cystic changes in the macula that develop in loratadine-related maculopathy. First, loratadine has direct toxic effects on Müller cells without disrupting the BRB. Changes in cellular metabolism cause intracellular fluid retention and swelling of these cells, which leads to the formation of intraretinal cysts.^17^^,^^18^ After cessation of loratadine therapy, there is partial or complete recovery of Müller cells and their normal function, accounting for the resolution of CME.

Retinal pigment epithelium (RPE) maintains retinal attachment by actively pumping water and electrolytes out of the subretinal space. Earlier research in 1991 reported that histamine stimulates both RPE phosphoinositide turnover and intracellular Ca^2+^ release through HR1.^30^ Recently, an *in vitro* study of a cell line from the human RPE (hRPE-YC) showed concentration-dependent increases in cytosolic Ca^2+^ concentrations after treatment with histamine and complete suppression of histamine-induced Ca^2+^ mobilization by the HR1 antagonist *d*-chlorpheniramine in hRPE-YC cells.^31^ However, substantial *in vivo* evidence is lacking to prove the hypothesis that interference of RPE pump function is involved in intraretinal water retention following long-term use of loratadine.

Other hypothesis suggests that subclinical disruption of the normal BRB might be caused by molecules with a molecular weight lower than that of fluorescein, which leads to fluid accumulation in the intracellular or extracellular space with no leakage on FFA. Nevertheless, histamine HR1 and HR2 stimulation of retinal vessels mediates BRB permeability.^32^ Inhibition of histamine activity with HR1 or HR2 antagonists returns BRB leakage to control levels despite uncontrolled diabetes.^33^^,^^34^ Therefore, damage to the BRB might not be a possible mechanism for loratadine-related CME in this patient.

As a nonprescription drug, the recommended dosing of loratadine for allergic rhinitis or conjunctivitis is 10 mg once daily or 5 mg twice daily, with no definitive limit in the course of treatment, and additional cases developing CME must be studied to establish a real association. When examining patients with macular edema, a history of drug intake should be considered, especially when there is no leakage observed on FFA. Further research into the mechanisms of the ocular adverse events resulting from loratadine might be helpful in highlighting this association.

## Funding

This study was funded by Tianjin Key Medical Discipine (Specialty) Construction Project.

## Authorship

All authors attest that they meet the current ICMJE criteria for Authorship.

## Patient consent

Consent for publication of this case report was obtained from the patient in writing.

## Declaration of competing interest

All authors declare no conflicts of interest.
